# Burring Machines

**Published:** 1871-07

**Authors:** 


### SELECTED ARTICLES.

---

### ARTICLE V.

### *Burring Machines.*

At a meeting of the Odontological Society of Great Britain, Mr. Harrington exhibited a machine to supercede the use of the hand drill. He said the principle of the machine first suggested itself to his mind some eighteen years since; after almost innumerable attempts, he succeeded in making it available for practice. About five years ago he had the pleasure of exhibiting one before the members of the Society, and very naturally as the inventor of the machine, he flattered himself that it would have been adopted by the profession, but in this he was disappointed. The chief objection to its use being that it was too noisy when in operation; he was bound, however, to say that, in his own practice he had never met with any complaint from his own patients on this score. Being anxious that his professional brethren might avail themselves of his invention, he had turned his attention to subduing the noise complained of, in which he had completely succeeded. The rapidity with which it worked would render it of great value to men in large practice; he calculated he could do as much in ten minutes with it, as with the ordinary hand drill he could accomplish in one hour, and at the same time with much less discomfort to the patient. It would be found principally valuable in two classes of cases—one where the lines of decay ran in a cruciform manner along the grinding surface of molar teeth; these cases were often but imperfectly stopped, owing to the difficulty of opening up the decayed line, but which could be effected with the machine under consideration in a few minutes,—the other, where it was found necessary to remove an amalgam stopping. Mr. Harrington illustrated, before the Society, the rapidity with which stopping could be removed. The machine could be kept perfectly under control while in use, by the thumb of the operator, and its speed could be regulated with the greatest nicety, or could be instantly stopped. With one winding it would work for two minutes. The present machine was superior to the former, not only from its noisleseness, but from the superior material of which it was made.

Mr. Fox said, as he had, through the courtesy of Mr Harrington, had the opportunity of thoroughly testing the capabilities of the machine, he felt bound to give the result of his experience with it, which might be briefly summed up in the remark that, having used it for some months, he should be sorry ever to be without it again. Like everything else, it takes a little time to get accustomed to it. When once the first difficulty is overcome, it becomes invaluable in the operating room. The apparent cumbersomeness disappears after a little practice, the very weight of the machine being an aid in the drilling. A hand drill only cuts on the half-turn forwards; during the back turn no advance is made; but with this instrument you cut all the time it revolves. Although the silent form was best, he did not find patients object to the noisy one—they invariably prefer it to the hand drill,, as being far more expeditious, but, in fact he had always found that patients would believe in what you believed in yourself.

The machine is wound up the same as a clock, and should never be allowed to run down without being checked by placing the thumb on the guide wheel.—*Transactions of the Odontological Society of Great Britain.*

---

